# ‘From a place of vulnerability’: Experiences of Voice Dialogue to explore self‐criticism

**DOI:** 10.1111/papt.70028

**Published:** 2025-12-12

**Authors:** Sarmini Indramohan, Richard J. Brown, Katie Reid, Matthew Pugh, Tobyn Bell

**Affiliations:** ^1^ Division of Psychology and Mental Health, School of Health Sciences University of Manchester Manchester UK; ^2^ Greater Manchester Mental Health NHS Foundation Trust Manchester UK; ^3^ Research Department of Clinical, Educational and Health Psychology University College London London UK

**Keywords:** chairwork, interpretive phenomenological analysis, low self‐esteem, qualitative, self‐criticism, Voice Dialogue

## Abstract

**Objective/Background:**

Voice Dialogue is a method used to explore and understand different parts of the self. It involves direct communication between a facilitator and aspects of the self to enhance awareness, understanding and differentiation from inner voices. This study aimed to understand how people with low self‐esteem (LSE) experience a Voice‐Dialogue session focusing on self‐criticism (or the ‘self‐critic’).

**Method:**

Nine individuals from a university setting underwent a single Voice‐Dialogue session that involved direct dialogue with their self‐critic and were subsequently interviewed about their experiences. The interview data were analysed using interpretive phenomenological analysis (IPA).

**Findings:**

Three group experiential themes (GET) were generated: theme 1, ‘transitioning between selves’, highlights the importance of moving between chairs and embodying the self‐critic; theme 2, ‘what makes the critic’, explores insights into the self‐critic's origins and function; and theme 3, ‘a change in relationships’, describes adaptations in participants' self‐to‐self relating.

**Conclusions:**

The findings suggest the Voice‐Dialogue method, as a stand‐alone intervention, has therapeutic utility in changing clients' relationship with their self‐criticism. The findings are contextualised within broader theory and literature, and clinical implications are discussed.

## INTRODUCTION

Voice Dialogue is a non‐pathologising method for differentiating, exploring and understanding different parts of the self (Stone & Stone, 1989). One part of the self that is addressed in Voice Dialogue is the self‐critic. Self‐criticism is a universal experience that acts as a vulnerability factor for a wide range of anxiety and mood disorders and is associated with global negative views of the self (Werner et al., 2019). This research explores the potential benefit of offering Voice‐Dialogue methods for people experiencing problematic self‐criticism in a non‐clinical population.

Voice Dialogue is a therapeutic method based on the concept that everyone is made up of numerous ‘selves’, each having their own perception of the world, personal history, emotional reactions and opinions on how to live our lives (Stone & Stone, 1989). These selves are organised into primary and disowned selves, which broadly map onto the more dominant and disowned parts of one's personality (Schnackenberg, 2023). The primary selves strive to help the individual meet core human needs (such as belonging) and be accepted in society so that they can succeed in life. Schnackenberg (2023) further explains that disowned selves are parts of the self that have been sidelined or repressed by primary selves as they are not accepted within one's culture and context. Stone and Stone (1993) suggest that the organisation of selves is determined during childhood and, despite adaptations made across the lifespan, the way in which ‘selves’ are constellated typically remains unchanged. As a result, the strategies used by a primary self to survive and cope in earlier circumstances may not be adaptive to new contexts and relationships, subsequently creating internal conflict and adversely influencing broader functioning and successful maturation (Corstens et al., 2012).

The Voice‐Dialogue approach facilitates an exploration of various selves to heighten self‐awareness, understanding and separation from inner voices. It also allows individuals to use the power and potential of their inner voices in an intentional and conscious manner (Chua et al., 2022).

In practise, Voice Dialogue utilises chairwork methods drawn from psychodrama (Moreno, 1948), which involve the use of chairs, the positioning of chairs and movement between them, to facilitate dialogue between parts of the self (Bell et al., 2021; Chua et al., 2022; Pugh, 2021). A Voice‐Dialogue session involves the participants changing positions in the room to embody a specific self. Upon switching chairs, the facilitator engages with this part by asking facilitative questions which generate insight into the self's function, content, origins, intentions and relationships with other parts of the self (Corstens et al., 2012; Stone & Stone, 1989). Upon completion, the client returns to their original chair and is supported to separate from the self‐part that has been embodied. Other parts of the self can then be identified and explored in a similar manner, using the same or additional chairs. At the end of the session the client is invited to adopt a witnessing position by standing alongside the facilitator and summarising what has occurred. Crucially, the Voice‐Dialogue facilitator does not challenge or pressure a self to change; instead, they model an attitude of curiosity and validation (Stone & Stone, 1989). Ideally, this process allows individuals to experience greater awareness, freedom and balance in how they engage with their various selves and the different ‘energies’ they embody (Schnackenberg, 2023). Research in Voice Dialogue has shown that it is an effective tool in fostering understanding and increased agency over specific voices or parts of the self, reducing related distress for people given a diagnosis of psychosis (Longden et al., 2021; Schnackenberg et al., 2018; Steel et al., 2020) and anorexia nervosa (Burnett‐Stuart et al., 2024; Chua et al., 2022).

Self‐criticism is a tendency to condemn, devalue and attack oneself (Gilbert et al., 2004). These negative evaluations take the form of thoughts and negative beliefs that are triggered in situations where there is a risk of failure or judgment (Whelton et al., 2007). Self‐criticism is also associated with negatively valenced self‐directed feelings, such as frustration, disappointment and disgust (Whelton & Greenberg, 2005). Self‐criticism is a universal experience that transcends culture, race, class and gender (Whelton & Henkelman, 2002). Research suggests that self‐criticism may be maintained by automatic and maladaptive coping strategies such as unhelpful dialogue with their self‐critical voice (e.g., reasoning with the self‐critic, weighing pros and cons, or refuting the self‐critic) (Dyson‐Horton, 2021), as well as perfectionism (Gilbert et al., 2006), self‐blame, overgeneralisation of failures or inappropriate emotional regulation (Kannan & Levitt, 2013). From a developmental perspective, self‐criticism is shaped by environments of chronic disapproval, criticism and/or conditional approval (Starrs et al., 2015). Additionally, self‐critical thoughts can be influenced by direct or indirect parental or societal rejections towards certain thoughts, behaviours or attitudes (Firestone, 1987).

Various forms of self‐criticism have been discussed in the psychotherapy literature (Bergen, 1995; Castilho et al., 2015; Driscoll, 1989). For instance, a common distinction has been made between two types: scathing self‐criticism, often referred to as ‘hateful’ or ‘persecutory’ self‐criticism, and more functional ‘corrective’ or ‘self‐improving’ self‐criticism (Gilbert et al., 2004). It has been suggested that the former tends to arise from the internalisation of toxic messages received in the past, such as from hostile caregivers or abusive individuals. In contrast, corrective self‐criticism aims to protect, improve or humble oneself through denigration (Bergen, 1995; Rafaeli et al., 2011; Simpson, 2019; Stone & Stone, 1989). More generally, self‐criticism has been identified as a transdiagnostic maintenance and vulnerability factor associated with various mental health conditions such as low self‐esteem (LSE; Thew et al., 2017), depression (McIntyre et al., 2018), disordered eating (Pascual‐Leone & Baher, 2023; Zelkowitz & Cole, 2019), post‐traumatic stress disorder (Harman & Lee, 2010), non‐suicidal self‐harm (Zelkowitz & Cole, 2019) and suicide (Cox et al., 2004; O'Neill et al., 2021).

Different therapeutic modalities treat self‐criticism in different ways. Cognitive therapy focuses on restructuring and reattributing self‐critical thoughts, helping individuals view these cognitions more objectively (Beck et al., 2024; de Oliveira et al., 2012). Emotion‐focused therapy (EFT) uses chairwork to create a dialogue between the critical and criticised parts of the self in order to clarify related primary emotions and needs, and foster a more helpful internal relationship (Greenberg, 2010; Shahar et al., 2012). Psychodynamic therapies focus on critical voices and dialogical narratives to help clients recognise and verbalise the attitudes of the self‐critic and explore their origins, followed by building on the client's confidence and self‐worth (Firestone, 1987). Compassion‐focused therapy aims to help clients develop skills to relate to the criticised and critical aspects of the self with compassion and work with the fears and threats that drive the critic (Gilbert, 2010). Schema therapy tends to link the critic back to its external origins, reduce its power via dialogical challenges, care for the vulnerable child that experince the criticism, whilst developing new 'healthy adult' competencies (Arntz & Jacob, 2017; Taylor et al., 2017). Across these therapies, the self‐critic is addressed by altering the way it is understood and processed by clients (Kannan & Levitt, 2013) and by teaching clients skills to relate to their self‐critic in new and more effective ways. Additionally, self‐criticism is often personified as a self‐critical voice (SCV) or referred to as the self‐critic (e.g., Gilbert, 2022; Kannan & Levitt, 2013) which can then be interviewed, enacted or observed from a distanced perspective (Pugh, 2024).

Despite the wide variety of therapies that address self‐criticism, studies have shown that the presence of self‐criticism reduces the effectiveness of evidence‐based treatments and is associated with poor treatment outcomes (Löw et al., 2020; Marshall et al., 2008; Rector et al., 2000), suggesting a need for innovative interventions. Several authors have highlighted the therapeutic importance of identifying the specific type of self‐critic the client struggles with (e.g., corrective or persecutory) as this can inform the case conceptualisation and guide the selection of appropriate interventions (Bergen, 1995; Simpson, 2019). Moreover, previous studies suggest that Voice Dialogue is a promising way to explore distressing parts of the self (Burnett‐Stuart et al., 2024; Chua et al., 2022).

To our knowledge, no study has yet investigated the use of Voice Dialogue in the context of self‐criticism. This study is the first to examine the experience of a Voice‐Dialogue session focusing on self‐criticism among individuals with LSE. Due to its exploratory nature, a qualitative methodology was used to provide a rich account of the experience of engaging with the self‐critic among individuals with LSE.

## METHOD

### Design

We used interpretative phenomenological analysis (IPA; Smith et al., 2022) as a methodological and interpretive framework for data collection and analysis. IPA provides a practical structure that weaves phenomenological descriptions and interpretive insight through the ‘double hermeneutic’ process (Smith et al., 2022). This process of shared meaning‐making of experiences matches the study's aim of exploring and interpreting participants' subjective, emotionally charged experiences. There is precedence for using IPA to explore the experiences of individuals' anorexia voices through the Voice‐Dialogue session (Chua et al., 2022), indicating the suitability of this framework for data collection and analysis.

### Ethics

This study received approval from the Research Ethics Committee of a university within Greater Manchester.

### Participants

Smith et al. (2009) recommend using a homogeneous sample to compare and examine experiences within a group sharing a similar context. Therefore, the researchers recruited individuals with LSE, who would be easily identifiable and representative of a specific group. Research indicates that LSE and self‐criticism are related (e.g., Gittins & Hunt, 2020). LSE is a negative perception of oneself (Brown, 2014), formed through negative evaluations of one's competency and acceptability (Mruk, 2013). Fennell (1997) developed a model of LSE that highlights the role of self‐critical thinking in maintaining negative thoughts and core beliefs about one's self‐worth. LSE and self‐criticism appear to be highly interconnected, creating a vicious cycle that perpetuates the deterioration of both conditions (Thew et al., 2017). Based on this strong connection between LSE and self‐criticism, the researchers focused their recruitment on individuals with LSE.

Participants were recruited via posters around the University and within the University Counselling Service (UCS). The inclusion criteria were current students or staff of the university who self‐identified as having LSE and spoke English. Participants were recruited on a first‐come, first‐served basis within a set timeframe. Nine participants completed the study. The sample size for this study was guided by Smith et al. (2022) and other comparable IPA studies (e.g., Chua et al., 2022). Table 1 summarises their demographic information. All identifiable information has been anonymised.

**TABLE 1:** Participant demographics.

Participant alias	Gender	Age	Ethnicity	LSE score	Self‐criticism score
P1: Sanjay	M	34	Indian	12	51
P2: Nick	M	20	White British	13	48
P3: Fatima	F	32	British Pakistani	11	Not completed
P4: Tom	Trans Man	25	White British	14	49
P5: Aruna	F	22	Indian	19	52
P6: Sarah	F	38	White British	24	49
P7: Alice	F	21	White	13	51
P8: Patrick	M	29	White British	21	46
P9: Lisa	F	41	Mixed	24	49

Participants completed two measures before the Voice‐Dialogue session. This information was gathered to describe the characteristics of the sample.

- The Forms of Self‐Criticising/Attacking and Self‐Reassuring Scale (FSCRS; Gilbert et al., 2004) was used to measure participants' level of self‐criticism, self‐hatred and capacity for self‐reassurance. The FSCRS is a reliable measure of self‐criticism (Baião et al., 2015).
- The Rosenberg Self‐Esteem Scale (RSES; Rosenberg, 1965) was used to measure positive and negative feelings about the self, thus providing a measure of self‐esteem within the sample. The RSES provides a reliable and internally consistent measure of global self‐esteem (Dhingra, 2013).

Participants were aged between 20 and 41 years old (mean = 29.11; SD = 7.66). Participant scores on RSES (*M* = 16.78, SD = 4.94) were lower than average compared to a non‐clinical sample of adults in the United States (*M* = 22.64, SD = 5.0) (Sinclair et al., 2010). Participants' FSCR scores were: ‘inadequate self’ (*M* = 30.38, SD = 3.90), ‘reassured self’ (*M* = 8.00, SD = 6.00) and ‘hated self’ (*M* = 10.88, SD = 4.21). When compared to non‐clinical samples, participants scored higher than average on IS (*M* = 17.72, SD = 8.29) and HA (*M* = 3.68, SD = 4.59) and below average on the RS measure (*M* = 20.27, SD = 5.77) (Baião et al., 2015).

### Procedure

The Voice Dialogue and interview sessions took place on different days (with the interview taking place no less than 7 days after the Voice‐Dialogue session). The Voice‐Dialogue sessionoccured in person, in rooms designed for therapy in a university building. Eight sessions were conducted by the fourth author and one session was conducted by the third author. All interview sessions were conducted by the first author. Both Voice Dialogue and interview sessions lasted between 60 and 90 min.

**FIGURE 1:**
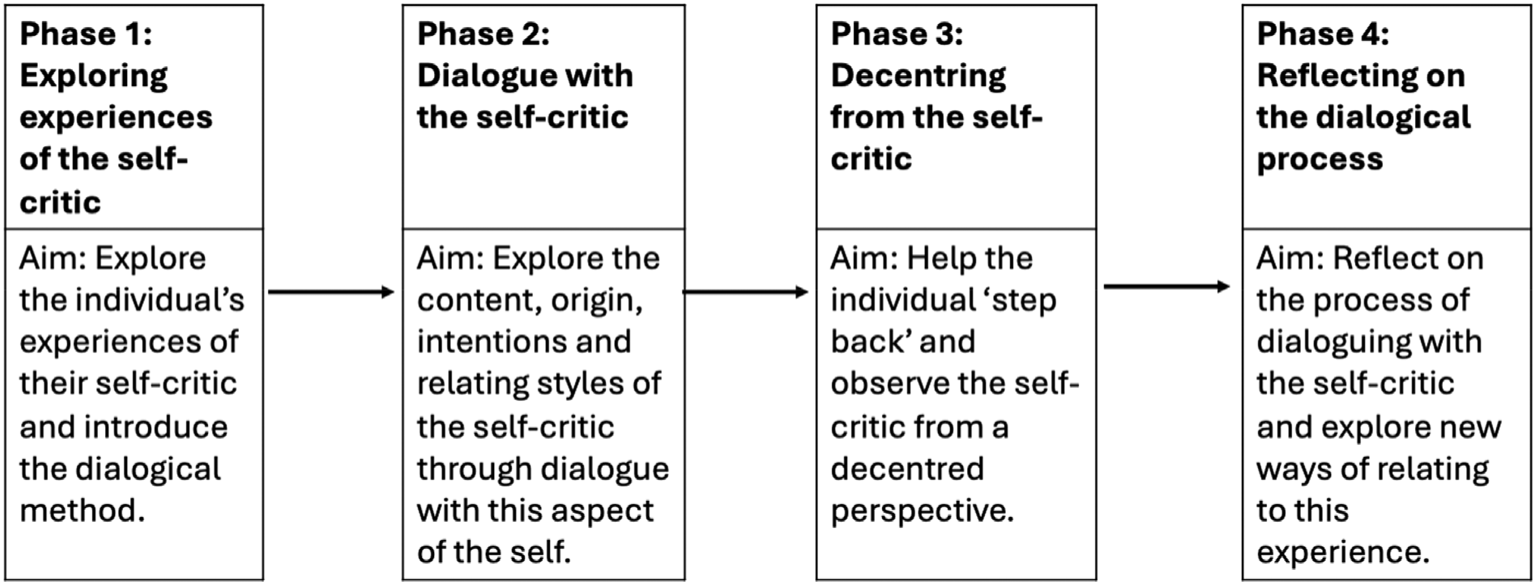
Structure for Voice‐Dialogue session.

Participants attended one stand‐alone Voice‐Dialogue session. The Voice‐Dialogue session had four main phases, all of which were included in each of the sessions offered. The Voice‐Dialogue session is summarised in Figure 1 (please contact the corresponding author for the full protocol). The topic guide was reviewed by an expert by experience, and the research team conducted a trial run of the guide. A semi‐structured interview schedule was developed in line with the study's aims. Examples of questions from the interview schedule are summarised in Table 2. Interview questions were open‐ended and looked at the overall experiences of participants and their experiences during the four phases of the Voice‐Dialogue session. A semi‐structured design was used flexibly for participants to describe and explore their experiences; the researcher then asked further questions to understand these in greater depth. The interview schedule was discussed with an expert by experience. Example questions from the interview schedule are presented in Table 2. The interview was audio‐recorded and recordings were transcribed by the researcher and pseudonymised before analysis (Table 1).

**TABLE 2:** Interview schedule.

Interview schedule and example of questions
Introductory Questions For example, Please tell me about your overall experience of the session Questions for Phase 1 For example, How did you find using chairs and moving positions during the exercise? Questions for Phase 2 For example, What was it like to act out your self‐critic in the exercise? Questions for Phase 3 For example, What was your experience taking a step back and reflecting? Question for Phase 4 For example, Has your experience during the exercise influenced how you think or feel about your self‐critic? General Reflective Questions: Are there any broader factors that influenced your experience of the session?

### Data analysis

Interviews were analysed using IPA (Smith et al., 2022). The first author analysed the data, according to the seven‐step IPA process detailed by Smith et al. (2022). Interview transcripts were read and re‐read multiple times to build familiarity with participants' accounts (stage 1). This was followed by line‐by‐line coding to develop initial exploratory notes (stage 2). Next, exploratory notes were summarised to develop experiential statements that represented units of meaning within the data (stage 3). Similar or related statements were connected and clustered together (stage 4) as personal experiential themes (PETS). PETS were themes that represented the experiential patterns and underlying meaning within and across individual data sets (stage 5). This sequence was repeated for each interview transcript (stage 6). Finally, PETS were collated and compared across participants to identify shared or differing experiences among participants (stage 7). This was synthesised into a narrative account for presentation. Direct quotations from the interview transcript have been provided so that readers can assess the reliability and credibility of the analysis (Dallos & Vetere, 2005).

### Reflexivity

Smith et al. (2009) acknowledge that the process of analysis is facilitated and complicated by the researchers' ideas and preconceptions (the double hermeneutic described above; Smith & Osborn, 2015). The researcher kept a reflective diary, noting down expectations and prior assumptions, to identify and manage how they shaped the data analysis and to also explore how they were changed or adapted in interaction with the data. For example, noting down the researcher's expectations and predictions of areas that participants might have found most interesting or difficult during the interviews. Once identified, the researcher was able to hold this in mind while analyzing the raw data and actively explore contradictions and variations. The research supervisor independently analyzed two transcripts to compare with the researcher's analysis to ensure themes were grounded in data and the analytic process was both systematic and transparent.

## RESULTS

The analysis generated three interconnected group experiential themes (GETs) related to embodying, understanding and forming a new relationship with the self‐critic. These themes and related sub‐themes are shown in Table 3.

**TABLE 3:** Summary of themes.

GET	PET	Participants per theme
1. Transitioning between selves	1.1. Movement and Separation 1.2. Embodying a new role	9/9
2. What makes the critic	2.1. Pain of the past 2.2. Underneath the ‘iceberg’—the true function of the self‐critic	8/9
3. A change in relationships	3.1. Meeting in the middle: new agency 3.2. A new compassionate relationship	8/9

### Theme 1: Transitioning between selves

#### Movement and separation

All participants commented on the usefulness of changing the spaces they occupied within the room. The movement between chairs was seen as a marker for ‘setting the scene’ (Sanjay) to get into the right mindset to embody their self‐critic. The change in physical positioning and perspective in the room was linked to an emotional shift and a distancing from their usual singular sense of ‘self’:

that physical distance that creates, that emotional distance to talk about myself from a third person's perspective. (Tom)

Like Tom, other participants described the process of moving chairs as a transitional phase that enabled them to embrace a different part of themselves. This experience also allowed them to talk about themselves in a decentred ‘third‐person perspective’. The distance gained from the original chair afforded participants the opportunity to reflect and envision themselves as separate and ‘other’.

Just the way it was positioned, it felt like an outsider maybe or sort of like just someone who is not actually in the conversation who was just sitting there. I guess because the self‐critic is in my head listening into my conversation but like, it's not part of those conversations, it's having a separate conversation with me. (Aruna)

Aruna illustrated how the chair designated for the self‐critic was positioned away from the occupied chairs of herself and the therapist, as though it were not meant to be included in the conversation, much like her self‐critic was an observer who scrutinised her daily experiences. Therefore, when she changed chairs to embody the self‐critic, she drew on these characteristics (e.g. the chair's positioning) to gain separation from her ‘self’. Like Aruna, other participants also noted how the physical characteristics of the chair they transitioned to (e.g. being stiff or taller than the others) took on metaphorical meaning and aided them in connecting with their self‐critic.

Through this physical movement through space between chairs, most participants reported experiencing emotional and physiological changes within themselves when they moved between seats and roles:

It was just like I went from like crying and being an emotional state and one chair to like distant sorry‐ distancing myself from that emotion in the other chair. (Fatima)

Fatima demonstrated how physical movement between chairs created a sharp distinction between selves, clarifying their impact and emotional character. Fatima's emphasis on ‘distancing myself from that emotion’ also highlights how movement between chairs fostered a sense of agency and efficacy over their emotion and immersion in parts of their self.

#### Embodying a new role

Participants were split over the ease with which they were able to embody their self‐critic. The majority of participants (6/9) felt surprised at how easily they were able to get into the role. For these participants, embodying the critic came naturally.

I thought it would be more difficult to do and I thought I would sort of keep slipping in and out of character, as it were, and I would be quite biased about it… I was quite surprised by how I just went into this kind of other character that I could sort of somehow access that I knew very well…. So it's kind of quite almost quite an eerie sort of experience to go into. (Sarah)

Similar to Sarah's experience, other participants commented on the ease with which they could embody the critic, which appeared indicative of their critic's strength and dominance. Sarah described her overall experience of embodying the critic as ‘eerie’, suggesting that this ease was tied to the critic taking over, rendering the experience haunting and strange despite its positive aspects. In contrast, another third of the participants (3/9) described embodying the critic as ‘trying to open a locked safe’ and expressed challenges in fully embodying the role:

When the questions were being asked, there were two things happening, one I was just thinking from a self‐critic perspective, but I was also thinking from my perspective. So, moving to a different chair was helpful in some aspect but not sure, I couldn't be like, er‐ couldn't embody the self‐critic 100%. (Sanjay)

Sanjay describes a push and pull of his attention, divided between the two parts of himself, where he could not fully embody the perspective of the self‐critic. Similarly, other participants found embodying their critic in isolation to be ‘annoying’ and ‘irritating’ (Nick), given the effort required to filter out thoughts and feelings from their other selves. One participant, Aruna, described the slow, searching, subjective process it took to embody the critic's role:

When I was first being, acting as the critic, it sort of I didn't really know where I was going at first… it was sort of like there was feeling in the dark for it” to “at some point I just felt like I was the critic, I felt like there was a very clear division between who I was and then who the critic was…and within that division, it felt very natural to feel what the critic was. (Aruna)

As Aruna described, participants felt a sense of division between the ‘self’ as a whole (as experienced in everyday life) and the ‘self’ as a specific part (as the critic). Aruna clearly experienced the switch and, once across the dividing line, felt this as a normal and ‘natural’ feeling.

### Theme 2: What makes the critic

#### Pain of the past

While in the role of the self‐critic, all participants were able to make connections to past experiences that shaped and influenced the content and nature of their self‐criticism. It appeared that their self‐critic was still ‘holding onto old values’ (Alice) from the familial and cultural contexts of their youth:

The self‐critic is impacted by so many different things like my gender, my sexuality, my culture, my background because I have seen, it's very indirect, but I have seen how women, in general, were treated so I'm a little more sensitive to those issues so everything little by little has impacted it. (Sanjay)

Sanjay spoke about growing up in a ‘queer‐ignorant’ environment, which led his critic to focus more on his mannerisms and how he interacted with others. Despite participants coming from various cultures and backgrounds, each commented on how societal, cultural or spiritual expectations and norms had shaped the formation and functioning of their self‐critic. Much like Sanjay's self‐critic, many participants discussed how class (e.g. coming from a ‘labourer’ background [Patrick]), gender (e.g. being a ‘girl’ [Aruna]), and spiritual beliefs influenced the focus of their self‐critic.

Through dialoguing as their self‐critic, participants gained insight into how negative experiences influenced the historic role and function of their self‐critic. Such experiences included receiving harsh comments about their appearance and behaviour, facing rejection from family or peers due to their sexuality or disability, and being pressured to work hard to avoid failures or ‘*falling short of those very high standards*’ (Lisa). Participants could also identify specific individuals in their lives (e.g. parents, relatives, friends and teachers) who contributed to these negative experiences and unmet needs. During the session, participants began to notice similarities between how they spoke as their self‐critic and the voice of individuals from their past, describing the self‐critic's speech as ‘learnt behaviour’ (Fatima):

I often hear like a lot of the criticism or like the unpleasant comments that people have told me…I kind of thought it was just exactly, that their voice is coming through, cause they have hurt and it's coming up to the surface again. (Aruna)

Aruna rationalised that when she felt ‘low’, she heard negative comments from her parents or teachers. Other participants shared similar experiences, where their self‐critic became much louder when a moment in their daily lives was linked to their past vulnerabilities and failures.

The majority of participants (7/9) experienced their self‐critic as being enmeshed with their sense of self:

the self‐critical voice in me is very much kind of mixed up with my own consciousness so it feels really hard to kind of tear it apart from my regular thoughts cause it comes so natural to me to be so critical of myself. (Alice)

Alice employed very raw language, such as ‘tear it apart’, and described her SCV as an ‘excessive force’ within her, indicating how intertwined her self‐critic and sense of self seemed to be. This sentiment was echoed in accounts from other participants, who portrayed their self‐critic to have become so closely intertwined with their sense of self, that it had consumed much of their ‘mental capacity’ (Nick). Consequently, participants found it more challenging to explore the history of their self‐critic during the exercise, as it seemed as though the self‐critic had always been a part of their lives and held the pain of their past.

#### Underneath the ‘iceberg’—The true function of the self‐critic

All participants seemed to reach a deeper level of understanding of the function of their self‐critic and the role it played in their lives. They came to the realisation that their self‐critic was a different part of the ‘self’:

So being able to compartmentalise and distance myself from that rhetoric and kind of rationalise it as being in a corner of my brain and being not a separate entity but a different section of the chemical soup, it's really been quite helpful. (Tom)

In completing the exercise, Tom was able to externalise his self‐critic to examine it as a separate ingredient within his usual thought processes (i.e., ‘the chemical soup’). This enabled him to identify and isolate his self‐critic and learn more about its role within his broader ‘self’ (for example, it is now positioned ‘in a corner’ rather than at the centre of the self). Such self‐distancing and self‐reflection allowed participants to assign roles for their self‐critic, with one group of participants (5/9) realising that their self‐critic acted out of anxious concern:

I think the most surprising thing was just that… both me and the self‐critic had like my best interest at heart or like they wanted to prioritise my health but they did it in different ways. (Fatima)

Through the Voice‐Dialogue session, Fatima realised that her self‐critic was ultimately concerned with her health and wellbeing. The realisation that her SCV was not just ‘a mean voice’ resulted in a strong emotional reaction for her both in the session and during the interview, indicating the impact of this new insight. Participants likened the role of the critic to that of an anxious parent, and identified similar concerns to those of their own parents. Participants therefore experienced their self‐critic as a form of safeguard to their emotional or psychological well‐being. Tom, for example, understood that self‐criticism helped him to ‘never be disappointed’ by imagining and preparing for worst‐case scenarios.

Another group (3/9) of participants realised that their self‐critic was acting as a protector linked to survival and self‐defense:

often times when I am self‐critical it is to preserve myself and to protect myself when it comes to a lot of my trauma and wanting to protect myself because I have been hurt by a lot of people around me so being self‐critical is kind of a way for me to distance myself from other people, and be kind of fend for myself in a way not have to rely on others. (Alice)

Alice describes her self‐critic as a form of self‐preservation against others, maintaining her autonomy and independence and linked to adaptive ways of coping with trauma. Whilst for some participants, the self‐critic acted as a protector against physical harm, for others, the self‐critic was protecting them from being bullied or criticised by other people, as they had been in the past. In understanding the true function of their self‐criticism, participants seemed to learn more about the depth at which their difficulties and worries had impacted them across their life span.

### Theme 3: A change in relationships

#### Meeting in the middle: New agency

The majority (5/9) of participants felt their new understanding of the self‐critic provided them with a novel sense of agency and possibility. This resulted in a new middle way: not attempting to get rid of the critic, but also not giving the critic complete authority. For Nick, this middle way offered him a new sense of power and balance:

a sense of regaining sort of power over your my own headspace, whereas before, I might have been feeling like the critic was taking up a huge amount of my mental capacity. (Nick)

As a consequence of the Voice‐Dialogue session, Nick and other participants identified a discrepancy and contradiction between the critic's aims and methods. Whilst the critic was understood to be driven by the goal of protection, participants also highlighted how its methods backfire, creating outcomes incompatible with this goal. Participants, therefore, concluded that whilst they agreed with their critic's aims, they differed in opinion about the way to achieve them. Such insights appeared to foster a willingness to disagree and disbelieve the content of their self‐criticism, and to adopt a new assertive tone to regain a degree of control. Some participants coined unique phrases (e.g. ‘dial it down’ [Sanjay] or ‘I'm trying’ [Lisa]) to speak back to their critic:

[The self‐critic] has a lot more meaning and content behind it, that is known to me by saying those words, I understand the deeper meaning behind them… and I said that I have been using day to day several times a day since that session so that was really good. (Lisa)

Like Lisa had described, these phrases were short and simple but held a special meaning to the participants and their self‐critics. They had the power to understand the self‐critic's history while also allowing space for the growth that the participants had experienced since the self‐critic's initial formation.

Some participants (4/9) believed that the understanding of their self‐critic helped them feel more confident and at ease with themselves:

I also feel a bit more confident because there's something about knowing that there's part of you that's pushing you so hard just because it wants to make sure you stay afloat, it feels almost like comforting in a way, like ‘aw you care about me’ I think that's quite important for me, because a lot of times in my life, I have felt not supported enough or like no one was really looking out for me enough. (Aruna)

Aruna expressed that she could make better decisions as she understood where her anxiety stemmed from. Other participants also found that by understanding the intentions behind their self‐critic, they could develop a new ‘emotional relationship’ (Sarah) with it and collaborate effectively. The simple act of understanding their self‐critics led to positive changes so profound that participants could sense a shift in power among the various parts of themselves.

#### A new compassionate relationship

The majority of the participants (7/9) identified a shift from having a hostile, bully‐victim dynamic with their self‐critic to a new, compassionate relationship. This change in self‐to‐self relating was largely due to the insight that their self‐critic was formed as an adaptive response to past fears and trauma and was directly linked to an underlying vulnerability:

I was relieved that this kind of part of myself that has been so nasty, and horrible for so long comes from a place of vulnerability just like I do, and I was sad that we had been misunderstanding each other for so long. (Tom)

Tom learnt that both he and his self‐critic held the same vulnerabilities, which led him to develop a feeling of sympathetic sadness towards the critic (and, by extension, himself)—feeling moved by a sense of purpose and concern. Other participants also described similar experiences in which they perceived the anger expressed by their self‐critic differently once they realised it stemmed from a place of fear and pain. Both realisations helped participants, ‘see a more human side of the critic’ (Sanjay) and empathise with it. This prompted the majority of participants (8/9) to desire a more compassionate relationship with their self‐critic:

looking after that‐ that part of myself basically, so the critics become another‐ I don't know how many I'm gonna end up with‐ but currently there's three of us and you know we're looking after each other and that's quite nice. (Sarah)

In acknowledging the self ascomplex and multiple (as 'we' instead of 'I'), Sarah began to appreciate the types of relationships between its contituent parts. There was a sense that these different parts contributed to her collective wellbeing by 'looking after each other', which she experienced as reassuring.

Sarah explained how she had become ‘quite compassionate’ (Sarah) towards her self‐critic, by adopting a gentler approach following the Voice‐Dialogue session. Likewise, other participants noted that understanding how their self‐critic related to other parts of the self and the surrounding world enabled them to ‘humanise’ (Sanjay) their self‐critic, which fostered a channel of communication that was ‘respectful’ (Sanjay) and ‘polite’ (Sanjay).

Some participants (5/9) shared that they learned how to be compassionate to themselves and their self‐critics from their therapist. The therapist either self‐disclosed their relationship with their own self‐critic, or modelled compassionate responses towards their self‐critics during the exercise:

[the therapist] also kind of also said the same that how I deal with it like ‘thank you for whatever you've done in the past but you're not serving me anymore’ but on some level I knew this, but then when you like put it into words and especially in the words how [therapist] said it, it really makes a difference I think that's something that I'm still processing but yeah it's still stayed with me. (Sanjay)

Sanjay reflected on how his therapist provided a potential script that articulated the right balance of compassion and assertiveness he aimed to capture when building this new relationship with his self‐critic. The therapist employed various methods, including self‐disclosure, as in Sanjay's case, or modelling the compassion and encouragement one might adopt towards their self‐critic. Alternatively, some participants shared that they enjoyed brainstorming compassionate ways of behaving or communicating that they could implement with their self‐critic, alongside their therapist. It seemed that participants drew from characteristics of their relationship with their therapist or the therapists' interactions with their self‐critic to build this compassionate relationship.

## DISCUSSION

The present study sought to understand how participants experienced a Voice‐Dialogue Session focused on self‐criticism (personified as ‘the self‐critic’). The results highlight the benefits of experientially exploring the self‐critic using chairwork and Voice‐Dialogue methods (i.e., speaking as the critic, moving to and from the self‐critic chair to separate). This finding aligns with a similar study (Chua et al., 2022), which utilised movement between chairs to help participants ‘witness’ and ‘speak up’ about their eating disorder voice, thereby facilitating more authentic dialogue between the voices during the session. The results of this study also highlight the valuable insights individuals can gain about their self‐critic, including its history and functions, through Voice Dialogue. This finding aligns with similar studies exploring this method (Chua et al., 2022; Schnackenberg et al., 2018). Finally, this study also showed that by engaging in the Voice‐Dialogue session, participants were motivated to change the way they relate to their self‐critic and felt more in control over their self‐critic.

Participant experiences from this study showed that the use of chairs provides a multi‐purpose tool (space and movement) to allow participants to separate and leave their original ‘self’ and embody their self‐critic (Pugh & Bell, 2020). The physical distance between the chairs, the position of the chair within the room, and the physical characteristics of each chair helped participants create a sense of self‐distancing and seperation. As suggested by Stone and Stone (2007), the original chair served as a reference point for the ‘self’; moving away from the original chair allowed for the exploration of new parts (e.g., developing a voice for the self‐critic). Participants shared that by accessing this new perspective in the room and adopting a different body posture,they began to feel their way into the role by connecting with their body sensations and physiological markers. This experience supports the notion that moving or leaving chairs facilitates a shift in affect and perspective (Bell et al., 2020). Through the movement from one chair to the next, participants in this study experienced a change in their emotional state (e.g. upset in the first chair to unemotional in the other) and physiological states (e.g. changes in posture, body language and tone), which they attributed to moving chair and role. Bell et al. (2021) suggest that changing states through intentional movement helps with the differentiation and organisation of different parts. Participants in a similar study exploring the anorexic voice also found the use of the movement between chairs helpful (Chua et al., 2022).

Participants had different experiences of embodying their self‐critic (e.g. some found it natural while others found it difficult and unnatural). One possible explanation is that their self‐critic was associated with distressing life events (Longden et al., 2012), and participants found it difficult or shameful to experience an unfiltered version of this aspect of themselves. Additionally, chairwork can also lead to higher emotional arousal (Bell et al., 2020; Lafrance Robinson et al., 2014). Hence, Stiegler et al. (2018) suggest that clients engaging in chairwork need to have sufficient skills to regulate their emotions, as this could hinder their ability to connect with and embody their self‐critic. Clinicians should approach Voice Dialogue and chairwork collaboratively with clients, respecting individual differences in capacity and preference instead of leading with an expert‐driven approach (Bell et al., 2020; Corstens et al., 2018; Steel et al., 2020). Bell et al. (2023) provide a third possible explanation for the difficulty participants experienced in embodying their self‐critic. In their study, they highlighted the importance of the therapeutic relationship in chairwork, showing how it helped participants feel comfortable exploring the different parts of themselves. In this study, participants had no prior rapport with their therapist and met them for the first time during the session. This may have prevented them from feeling entirely comfortable accessing their self‐critic with the therapist or fully engaging with chairwork. Despite their initial reservations and the variation in the degree to which participants felt they embodied their self‐critic, all participants felt that they gained a deeper insight and understanding through the experiential process of the Voice‐Dialogue session. This is consistent with other clinical groups that have also used Voice Dialogue to explore voice‐like experiences (e.g. individuals diagnosed with psychosis, anorexia or body dysmorphic disorder: Schnackenberg & Schnackenberg, 2023; Chua et al., 2022; Longden et al., 2021; Schnackenberg et al., 2018).

Participants indicated that societal, cultural and spiritual norms and expectations had influenced the creation of their self‐critic. This is consistent with the theoretical underpinnings of Voice Dialogue, where primary and disowned selves are governed by rules that were prevalent in childhood (Stone & Stone, 1989, 2007). Most participants imagined their self‐critic to be an amalgamation of the people in their lives who have been critical to them. All participants commented on how their Asian, European or Western cultural norms and expectations, no matter how outdated, formed the ‘rule book’ for their self‐critic. Firestone (1987) explains this experience as the self‐critic being influenced by parental or societal rejections towards certain thoughts or attitudes. Research with other clinical populations, like those with eating disorders, also suggests that childhood emotional abuse is positively linked to the power of the eating disorder voice (Pugh et al., 2018). Additionally, when core negative memories, past vulnerabilities or unmet needs are triggered in participants' daily lives, their self‐critic is activated (Dyson‐Horton, 2021). The current study supports the notion that in such instances, the self‐critic becomes upset or agitated, demanding more power so that it can help protect the individual from experiencing the pain of such vulnerable moments. This aligns with literature highlighting the underlying fears and supportive functions of some types of self‐critic (Bergen, 1995; Kellogg & Garcia Torres, 2021; Simpson, 2019). Hence, it is important to formulate the self‐critic within a developmental framework to understand its origins, as this seems to play a catalytic role in its formation (Corstens et al., 2012). Voice Dialogue, which focuses on the self‐critic, could be used explicitly for this focus, as a form of assessment or socialisation to help understand the influence of the clients' past on their SCV in the present.

Participants in this study recognised that their self‐criticism has varying functions, many of which centre on self‐preservation or threat management. This variation in the self‐critic's functioning aligns with previous research, suggesting that the self‐critic can operate on a spectrum from anxiety‐based self‐improvement to anger/disgust‐based self‐hatred (Castilho et al., 2015; Gilbert et al., 2004). Participants gained ‘action insights’ into the function of their self‐criticism by answering the Voice‐Dialogue questions in the role of the self‐critic (Stone & Stone, 1989, 1993). This study also supports the need to assess and differentiate types of self‐criticism using methods such as Voice Dialogue to conceptualise the self‐critic and guide intervention (Bergen, 1995; Kellogg & Garcia Torres, 2021; Simpson, 2019). This exploratory orientation (experiencing, acknowledging and understanding the critic) was seen as an essential part of the three therapies (cognitive, psychodynamic and emotion‐focused therapies) reviewed by Kannan and Levitt (2013). Whilst some authors have questioned whether speaking to, from or ‘about’ the self‐critic in this way might unhelpfully reify this pattern of self (Fahy, 1988), this was not the case for participants in this study. Such experiential exploration of the self‐critic ultimately appeared to provide a greater capacity for adaptive self‐reflection and integration, as clients focused on the role of this part within the broader self‐system. This finding echoes previous research on chairwork methods and self‐criticism (Bell et al., 2021). Notably, participants in this study were open and receptive to the concept of self‐multiplicity and reported no difficulties in considering how different parts of the self‐functioned in a modular manner with different roles or functions; similar experiences of Voice Dialogue have been reported elsewhere (Chua et al., 2022).

Like previous studies (e.g., Burnett‐Stuart et al., 2024), this paper demonstrates that Voice Dialogue also provides valuable insights into the nature of certain aspects of the self. Through this process of embodying their self‐critic, participants realised that despite its protective or defensive function, their self‐critic had either stopped serving its purpose, had become too powerful or was inflexible/unhelpful in pursuing its aims. This insight aligns with various frameworks for understanding self‐criticism: as a rigid and imbalanced internal relationship between parts of the self, shaped to meet the expectations and demands of important and powerful others from the past (e.g. Hermans & Dimaggio, 2004). Some participants realised the self‐critic was related to the maintenance of their LSE and negative view of themselves. Stone and Stone (1993) suggest that the self‐critic, in its attempt to protect clients from facing negative evaluation and rejection, will, for example, not allow them to feel successful or proud of things that they have accomplished. This leads to clients being chronically stuck in a loop of half‐praise or dismissed achievements, which can influence self‐esteem.

Changing the way the person understands and processes the self‐critic can help resolve their conflicts with it (Kannan & Levitt, 2013). Through a single Voice‐Dialogue session, participants were able to change their perspective on their self‐critic, seeing it as concerned and somewhat caring in its efforts. For many, the session was seen as a turning point in building a new relationship with the self‐critic. One of the ways participants learnt to relate differently to their self‐critic was with compassion, which has been shown to be effective in managing self‐criticism (Wakelin et al., 2022). In viewing their self‐critic with compassion, participants were open to listening to the self‐critic's fears and acknowledged the painful history linked to its origins. Participants also identified new experiences of self‐agency when engaging assertively with the critic. This is consistent with Voice‐Dialogue ideas about developing awareness of different parts to build choice and agency in how individuals relate to the various aspects of the self‐moving forward (Stone & Stone, 1989, 1993).

Participants in this study commented on how their therapist interacted with their self‐critic and how their curiosity, acceptance and compassion acted as a model for their own self‐to‐self relating. This aligns with Voice‐Dialogue literature, which suggests that facilitators should not teach specific skills but instead model ways of relating that clients may adopt (Stone & Stone, 1989). Bell et al. (2023) have also demonstrated how clients use their therapist as a compassionate model to guide how they could be compassionate to themselves during chairwork. Participants in the study also identified the importance of therapist self‐disclosure, which appeared to give permission for the client to explore and experiment with new forms of self‐relating.

### Limitations and future research

This study has several limitations. Whilst the majority of the participants scored below the cutoff for clinically significant LSE on the RSES, four participants scored higher than this threshold. The sample used in this study may therefore limit the validity and generalisability of the findings for an LSE population, despite all participants identifying themselves as having LSE. Notably, the scoring on the RSES has been applied differently in the literature, with the possible scores of each item ranging from either 0 to 3 or 1 to 4, despite using the same numerical cutoff for LSE (score ≤ 15) (Sinclair et al., 2010). This study followed the example of research focusing on the UK university student population (Dhingra, 2013), which scored items from 1 to 4, potentially leading to higher estimations of self‐esteem when compared to research using a 0–3 scoring system.

This study was conducted with a mixture of students and staff from the university. This is helpful in terms of understanding how the Voice‐Dialogue session can be adapted to people from various age groups and roles. However, it might be helpful to focus on specific demographics to identify more age‐specific themes or experiences. The study was exploratory, and participant experiences were collected within a week of the Voice‐Dialogue session, helping to gain immediate insight into the learning and experience of the session. It might be beneficial to investigate the longevity of these experiences by conducting an interview a few months after the session to see if participants still hold the same insights. Although a single‐session format was chosen for the purposes of this research, it would be helpful to explore if participants might have different experiences if they had a longer course of Voice‐Dialogue work. Future research could empirically examine the effectiveness of the Voice‐Dialogue sessions through quantitative pre‐ and post‐intervention measures. Alternatively, task‐analytic studies could be conducted to clarify aspects of the Voice‐Dialogue session that generate change (Pascual‐Leone et al., 2014).

### Clinical implications

Previous studies exploring Voice Dialogue have looked at more complex mental health conditions like anorexia nervosa (Chua et al., 2022) and psychosis (Schnackenberg et al., 2018). Whilst these conditions may include self‐criticism, this is the first research to focus exclusively on the self‐critic using the Voice‐Dialogue approach.

The findings suggest that Voice Dialogue is a well‐tolerated and well‐received intervention when delivered in only a single session. The research adds to the literature on chairwork (Pascual‐Leone & Baher, 2023) and supports prior findings on the role of movement to foster separation from problematic parts of the self (Bell et al., 2021). In terms of assessment, Voice Dialogue can provide valuable insights into the nature of the self‐critic (e.g., its origins, content, functions and underlying concerns). By gaining a deeper understanding of the type of self‐critic a client struggles with (such as whether it is corrective or persecutory) therapists can create more nuanced case conceptualisations (e.g., Bergen, 1995). From an intervention perspective, Voice Dialogue can help clients develop a new, therapeutic means of relating to self‐criticism, and one that fosters increased self‐compassion more generally. There is the potential that Voice‐Dialogue methods could be used in either of these functions to complement established, evidence‐based models that include self‐criticism, such as Fennell (1997). As the presence of self‐criticism can negatively impact the efficacy of evidence‐based treatments and protocols (Löw et al., 2020), it may well be beneficial to offer a limited number of Voice‐Dialogue sessions to target self‐criticism before engaging with such treatment or in conjunction with these treatments.

The study also adds to the growing literature on the therapeutic applications of Voice Dialogue (Burnett‐Stuart et al., 2024; Chua et al., 2022; Longden et al., 2021; Schnackenberg et al., 2018; Steel et al., 2020), as well as the benefits of compassion‐focused and experiential interventions for addressing self‐criticism (Gilbert, 2009; Shahar et al., 2012; van Maarschalkerweerd et al., 2021; Wakelin et al., 2022). Finally, it is worth noting that this study recruited participants from diverse backgrounds and ethnicities, providing insights into the nature and content of self‐criticism across different cultures. The findings suggest that Voice Dialogue is an adaptable therapeutic tool that holds promise for clients from diverse backgrounds.

## CONCLUSION

In summary, this preliminary study suggests that Voice Dialogue helps individuals gain insight into the nature, functions and origins of self‐criticism. This, in turn, offers valuable opportunities for increased self‐understanding and compassionate self‐relating. The core processes of Voice Dialogue, such as externalisation and movement between chairs, also helped participants create distance between themselves and their self‐critic, which allowed them to be more intentional about how they related and engaged with this aspect of their self. All participants reported that the single‐session nature of the intervention was acceptable and beneficial, suggesting this method might be used in a time‐limited application or integrated into longer courses of treatment (as a part of formulation or intervention, or both). Future studies exploring the effectiveness of the Voice‐Dialogue session are needed to validate the usefulness of this method for addressing self‐criticism in clinical populations.

## AUTHOR CONTRIBUTIONS

**Tobyn Bell:** Conceptualisation (lead); data curation (equal); formal analysis (supporting); investigation (equal); methodology (supporting); project administration (supporting); resources (equal); supervision (lead); visualisation (equal); writing—original draft preparation (supporting); writing—review and editing (equal). **Richard Brown:** Methodology (supporting); supervision (supporting); validation (supporting); writing—original draft preparation (supporting); writing—review and editing (supporting). **Sarmini Indramohan:** Conceptualisation (supporting); data curation (equal); formal analysis (lead); investigation (equal); methodology (lead); project administration (lead); resources (equal); visualisation (equal); writing—original draft preparation (lead); writing—review and editing (equal). **Matthew Pugh:** Methodology (supporting); writing—original draft preparation (supporting). **Katie Reid:** Investigation (supporting).

## CONFLICT OF INTEREST STATEMENT

There is no conflict of interest. The research was undertaken as part of a clinical doctorate course.

## Data Availability

The data that support the findings of this study are available on request from the corresponding author. The data are not publicly available due to privacy or ethics restrictions.
